# Learning strategies, study habits and social networking activity of undergraduate medical students

**DOI:** 10.5116/ijme.576f.d074

**Published:** 2016-07-17

**Authors:** Andrea Bickerdike, Conall O'Deasmhunaigh, Siun O'Flynn, Colm O'Tuathaigh

**Affiliations:** 1 School of Medicine, University College Cork, Brookfield Health Sciences Complex, College Road, Cork, Ireland

**Keywords:** Learning strategy, study habits, social media, academic performance, mode of entry into medicine

## Abstract

**Objectives:**

To determine learning strategies, study habits, and online social networking use of undergraduates at an Irish medical school, and their relationship with academic performance.

**Methods:**

A cross-sectional study was conducted in Year 2 and final year undergraduate-entry and graduate-entry students at an Irish medical school. Data about participants’ demographics and educational background, study habits (including time management), and use of online media was collected using a self-report questionnaire. Participants’ learning strategies were measured using the 18-item Approaches to Learning and Studying Inventory (ALSI). Year score percentage was the measure of academic achievement. The association between demographic/educational factors, learning strategies, study habits, and academic achievement was statistically analysed using regression analysis.

**Results:**

Forty-two percent of students were included in this analysis (n=376). A last-minute “cramming” time management study strategy was associated with increased use of online social networks. Learning strategies differed between undergraduate- and graduate-entrants, with the latter less likely to adopt a ‘surface approach’ and more likely adopt a ‘study monitoring’ approach. Year score percentage was positively correlated with the ‘effort management/organised studying’ learning style. Poorer academic performance was associated with a poor time management approach to studying (“cramming”) and increased use of the ‘surface learning’ strategy.

**Conclusions:**

Our study demonstrates that effort management and organised studying should be promoted, and surface learning discouraged, as part of any effort to optimise academic performance in medical school. Excessive use of social networking contributes to poor study habits, which are associated with reduced academic achievement.

## Introduction

There are numerous determinants of academic success in medical school, including prior academic achievement, personality traits, and individual differences in cognitive strategies employed during learning.^1^^,^^2^ A 25-year retrospective study conducted in a UK medical school concluded that high grades in second-level chemistry and biology examinations were predictors of later academic success in medical school.^3^ Similarly, high examination grades at second-level education were also deemed predictive of academic success in a Croatian study.^4^ Conversely those with evidence of less than optimal second-level and early undergraduate academic achievement are more likely to drop out of medical school.^3^^, ^^5^^, ^^6^

Certain personality traits have also been shown to correlate positively with academic success; for example, conscientiousness has been identified as a positive predictor during the preclinical years of medical school, even after controlling for previous academic performance.^2^  In relation to cognitive factors, it has been suggested that the predictive power of learning strategies is under-researched in the context of medical education,^1^ with some researchers concluding that students’ preferred learning strategy accounts for 49% of the variance in examination results amongst pre-clinical medical students.^7^

The nomenclature and terminology employed in the literature to describe the mode of learning that students adopt in higher education is diverse; “learning strategies”,^1^^,^^8^ “approaches to learning”^9^ and “learning strategies”^10^^, ^^11^ are terms that have been used in an interchangeable manner. In the present paper, we use the term “learning strategy” to define the approach that students take in order to learn their course material. Three broad learning strategies applicable to education in a general sense, and validated in medical students and practicing physicians, have been defined in the literature.^10^ Firstly, a deep learning strategy, where the student strives to achieve an intricate understanding of their subject matter. Secondly, a surface strategy, where students rote learn without necessarily understanding the material, and finally, a strategic approach, where students are motivated by a desire for academic success and consequently modify their learning to align with mode of assessment. The surface approach, which focuses on reproduction of rote learned material, is associated with a poor academic outcome.^12^^-^^14^ A deep learning strategy leads to the greatest level of academic understanding, but there is ongoing debate regarding how well it is correlated with superior examination results.^13^^, ^^15^ One study demonstrated a negative effect of the deep learning strategy on first year examination results in medical school, suggesting that it has to be combined with an ability to be pragmatic and organised in one’s learning in order to be beneficial in terms of exam results.^16^ Numerous self-report questionnaires have been developed to evaluate learning strategies, one of which is the Approaches to Learning and Studying Inventory (ALSI) which was developed in 1983 by Professor Noel Entwistle.^17^ This tool has been employed in multiple educational contexts and diverse populations,^18^^,^^19^ and has been validated in a cohort of medical students,^13^ although the latter study was conducted in a limited sample of students during the early years of the medical curriculum.

Choice of learning strategy is influenced by many factors including demographic characteristics, conceptions of learning, and contextual factors of the learning environment.^14^^,^^15^^,^^20^ Gender differences have been explored previously in relation to preferred learning strategies, with women reported to be more likely to employ a deep learning strategy.^16^ However, educational factors including mode of entry into medical school (i.e. undergraduate-entry vs. graduate-entry programmes) have not been studied extensively in relation to learning strategy preference. Both groups may differ in their learning strategy preferences as they seem to differ in their motivation to study medicine.^1^ It has also been demonstrated that graduate-entrants may outperform undergraduate entrants with respect to their overall academic achievement, especially in clinical assessments.^21^^,^^22^

International surveys which have focused on reasons for underachievement at third-level have concluded that second-level education may not promote the development of adequate study skills required for success at third-level. ^23^^, ^^24^  A qualitative analysis of semi-structured interview data showed that struggling medical students admitted to employing poor study habits; these included focussing on the wrong material and failing to designate adequate time for revision prior to written exams. ^25^ In medical education, time management has been shown to positively correlate with academic success in the first semester of an integrated curriculum.^26^ With regard to remediation for struggling students, time management has been the focus of study-skills interventions, yielding conflicting results.^27^ Additionally, the impact of the modern phenomenon of online social networking (OSN) on study habits and time management  is the subject of recent research; a number of studies have reported a detrimental effect of time spent online networking with respect to examination results.^28^^-^^31^                                                                              

Recognising the putative advantage of adoption of specific learning strategies to improve academic performance in medical education, this study will investigate whether there is a relationship between learning strategy, study habits (time management style, online social networking during study), and academic success in this population. These potential relationships will also be examined against the backdrop of academic (i.e. mode of entry into medical school) and demographic variables. This evaluation may assist the medical school in identification of students who are at risk of developing unfavourable approaches to academic tasks, and who may need additional direction or assistance to achieve their academic potential.

## Methods

### Study design and setting

A descriptive, cross-sectional study design was used. This study was carried out in the School of Medicine at University College Cork, Cork, Ireland. The study sample comprised medical undergraduates enrolled in the 5-year undergraduate-entry (UG) and 4-year graduate-entry medicine (GEM) programmes at University College Cork. Specifically, the study population consisted of two cohorts of Year 2 UG, Year 2 GEM, Year 4/Final Year GEM, Year 5/Final Year UG medical students. Curricular frameworks for both UG and GEM courses follow a spiral systems based structure with emphasis on case based learning and small group teaching. UG and GEM cohorts receive conjoined teaching at many points and are merged for the last two years of the course. For each programme year, the end-of-year grade is based on performance across several modules, with continuous assessment and comprehensive examinations typically employed for each module. Ethical approval was obtained from the School of Medicine Research Ethics Committee and the Research Ethics Committee of the Cork Teaching Hospitals. The study was exempt from consent requirements as the data met the requirement for de-identification as defined by Clinical Research Ethics Committee of the Cork Teaching Hospitals.

### Study measures

Two strands of data collection were employed in this study. The first comprised the collection of data on learning strategies, as measured by the ALSI,^32^ and study habits data from study participants. The second strand was the collection of year score percentage marks in the same students, in order to correlate learning strategies and study habits with academic success.

Measurement of Study Habits: Data related to study habits were collected from participants using several newly-devised survey items. Questions were of a multiple choice format, and collected data on participants’ usual study location (‘library’ vs ‘home’), preferred academic resource (‘textbooks’, ‘books’, ‘lecture notes’, ‘handwritten notes’) and the use of OSN websites during study time (‘never’, ‘sometimes’, ‘often’). Students were also asked to indicate whether they employed a “consistent” (‘consistently throughout the academic year, with a mild increase in study in the weeks leading up to exams’) vs “cramming” (‘huge amounts in the weeks leading up to exams, with relatively little study throughout the academic year’) time management strategy during their studies. As well as asking participants to indicate their year of study, this section of the questionnaire also collected demographic information on the participants, including age, gender, nationality and mode of entry into medicine.

Approaches to Learning and Studying Inventory (ALSI): The second part of the questionnaire collected data on learning strategies, using the 18-item version of the ALSI.^32^ The inventory is composed of four subscales: Surface Approach, Monitoring Studying, Deep Approach, Effort Management, and Organized Studying. Using a 5-point Likert scale, participants chose the answer which they felt best represented the extent to which a statement was true of them at that particular time (1=not at all true of me, 5=very true of me). Scores were obtained by averaging the Likert scaled responses for each of the 2-6 questions relative to each subscale, resulting in a score for each of the five variables. Reported confirmatory factor analysis (CFA) resulted in a 4-factor model (deep approach, surface approach, monitoring studying, effort management/organized study).^17^ Internal reliability by factor using Cronbach‘s α was as follows: Deep approach, 0.66; Surface approach, 0.64; Monitoring studying, 0.66; Effort Management, 0.34; Organized Studying, 0.72; and the merged factor, 0.70=Effort Management/Organized Studying. There was no time limit for completion of the questionnaire, but it took an average of 6 minutes to complete.

Measure of Academic Achievement: Year scores from the preceding academic year were used to correlate study habits and learning strategies with academic success.

### Data analysis

All continuous scale data sets were tested for normality prior to the selection of statistical tests. Year score percentages for each individual year group were compiled to form a single data set, which was deemed to be normally distributed using the Kolmogorov-Smirnov test (Z = 0.06, p = 0.08). This justifies our choice of independent samples t-test to compare mean year score percentage according to gender, mode of entry (UG and GEM students), and ‘consistent’ vs. ‘cramming’ time management strategy. In order to determine the effect of social networking during study time on year score percentage, one-way ANOVA was used. The chi-square test of independence was used to examine the relationship between each of the following factors: gender, mode of entry into medicine, and time management.

The results of the ALSI were not normally distributed; therefore non-parametric tests were used. Cronbach’s α reliability coefficient was calculated for each factor on the inventory to determine its internal consistency in our study. The Mann-Whitney U test was used to compare scores for each of the learning strategy between gender groups and between UG and GEM students. To correlate each learning strategy with year score percentage, Spearman’s rank correlation coefficient was used.

Multivariable linear regression analyses were conducted to examine the relative influence of categorical and continuous scale independent variables (each ALSI factor, gender, age, year of programme, mode of entry into medical school, time management strategy) on year score percentage.

## Results

376 students returned fully completed questionnaires, with response rate across each of the groups as follows: UG Year 2 – 54%; GEM Year 2 – 51%; UG Final Year – 22%; GEM Final Year – 19%. The difference in response rate between Year 2 and Final Year reflects increased number of students completing core rotations in off-site teaching hospitals or abroad. Ages ranged from 18 to 38, with a mean age of 22.34 (standard error of mean, SEM=0.17). Females comprised 55.3% of the total sample, and the breakdown according to nationality was as follows: Irish, 56.6%, North American, 11.2%, Asian, 30.6%, other, 0.5%.

### Study habits

Over half of the total student sample (52.9%) reported studying primarily in the library, 40.4% studying most often at home, and 7.1% of students stated they divide their study time equally between the library and home. No significant difference in mean year score percentage was observed between the three study locations (F _(2, 342)_ = 0.85, p = 0.43). 47.1% of the sample identified themselves as using a “cramming” (“huge amounts in the weeks leading up to exams”) time management strategy, with 49.7% reporting a “consistent” (“consistently throughout the academic year”) strategy. The latter time management strategy was associated with higher mean year score percentage (‘consistent’ vs.  ‘cramming’, 72% vs. 63.3%, t _(349)_ = 1.98, p < 0.05). No effect of medical programme or programme year was observed in relation to time management strategy (all p > 0.05), but females were significantly more likely to report a ‘consistent’ strategy (χ^2^ = 3.15, p < 0.05).

The majority, 93.6% of our sample, reported engaging in OSN. Facebook was the most popular social network (99.3%) among those engaged in OSN. When asked to specify how much time they spent on an average day actively using OSNs, the following usage pattern was observed: 0-30 min (31.9%), 30-60 min (30.9%), 1-2 hrs (21.0%), 2-4 hrs (8.2%), 4+ hrs (1.3%). Focusing specifically on whether OSN activity coincided with study time, 31.4% stated they use OSNs “often”, 55.9% stated “sometimes” and 10.1% of students stated “never”. Variation in year score percentage was not found to be dependent upon frequency of OSN use during study time (p > 0.05), and no significant difference was found between undergraduate and graduate entry students in relation to frequency of OSN use during study time (p > 0.05). Females were, however, more likely to report “never” using OSNs during study time (χ^2^ = 7.08, p < 0.05), and final year students were more likely to report “often” using OSNs during study relative to second year students from both programmes (48% vs. 29%; (χ^2^= 10.00, p < 0.01). A “cramming” time management strategy was associated with increased time spent using OSNs during study time (χ^2^ = 21.54, p < 0.01).

### Learning strategies

The ALSI was used to assess learning strategies. Student mean scores for each of the four factors, along with their respective Cronbach’s α coefficients, are shown in Table 1. In order to facilitate comparison between the mean scores, each mean numerical score is also expressed as a percentage of the maximum possible score for that factor.

**Table 1:** Descriptive results for the 4 ALSI factors in the study population

The 4 ALSI factors	Number of Questions	Mean	Mean as % of Max.	SEM	Cronbach’s α
Deep approach	6 Questions	19.84	66.10	0.22	0.71
Surface approach	4 Questions	9.57	47.85	0.15	0.64
Effort management /Organised studying	4 Questions	13.02	65.10	0.18	0.74
Monitoring studying	4 Questions	13.70	68.50	0.15	0.58

T-test or Mann-Whitney U analyses, where appropriate, revealed no significant gender differences in scores for any of the four factors (surface approach, deep approach, effort management/organised study, monitoring studying, all p > 0.05). However, UG students were statistically more likely to demonstrate a surface approach than their graduate-entry peers (U = 9303.5, z = 3.75, p < 0.01); UG students scored a mean score of 9.89 (± 0.17, SEM) vs. 8.62 (± 0.29, SEM) for GEM students. In contrast, GEM students were statistically more likely to demonstrate a monitoring studying strategy than their undergraduate-entry peers (U = 10345, z = 2.55, p = 0.01); GEM students scored a mean score of 14.43 (± 0.27, SEM) vs. 13.44 (± 0.18, SEM) for UG students. No programme differences were found in relation to deep approach or the effort management/organised study factor scores (both p > 0.05).

Table 2 provides a correlation matrix of the relationship between each of the learning strategies and year score percentage.  A surface approach learning strategy was found to be negatively correlated with year score percentage (r_s_ = -0.26, p < 0.001). Year score percentage was also found to be positively correlated with the management/organised studying (r_s_ = 0.29, p < 0.001) and monitoring studying strategy (r_s_ = 0.20, p < 0.001). As indicated in Table 2, surface approach was negatively correlated with scores across each of the other learning strategies, whereas each of the other three learning strategies was highly positively correlated with one another.

**Table 2:** Correlations between ALSI factors and year percentage score in the study population asterisk

ALSI factors	Surface approach	Monitoring studying	Effort management / Organised studying	Total year score (%)
Deep approach	- 0.25^*^	0.62^*^	0.31^*^	0.07
Surface approach	-	- 0.28^*^	- 0.35^*^	- 0.26^*^
Monitoring studying	-	-	0.41^*^	0.20^*^
Effort management / Organised studying	-	-	-	0.29^*^

*p < 0.01

### Multiple regression modelling for prediction of academic achievement

Multiple linear regression analysis was performed using the four ALSI domains, two study habit variables (‘consistent’ vs ‘cramming’ strategy), use of online social media during study (‘never’, ‘sometimes’, ‘often’), and four demographic/educational variables (age, sex, year of programme, mode of entry into medicine) as independent variables and year score percentage as the outcome variable (see Table 3). A poor time management approach to studying (i.e. “cramming”) and an increased tendency to employ the surface approach to learning was associated with decreased year score percentage. In contrast, increased use of the effort management/organised studying strategy was associated with improved academic performance.

## Discussion

The results of the present study demonstrate the detrimental effect of the surface learning strategy, and the beneficial effect of a strategic, organised approach to learning in a medical education context. In relation to demographic and educational variables (i.e. gender and mode of entry into medicine) which have been shown to influence academic achievement in medical school, we have built on existing research by not only comparing academic performance, but also by specifically comparing learning strategies between the groups. We have shown an increased prevalence of the undesirable surface learning strategy amongst UG vs. GEM students. With regard to study habits, we have identified time management as a skill which is poorly mastered by 47% of our medical student sample, and frequent use of online social networking sites is one of the factors we identify as contributing to this deficiency.

**Table 3:** Results of linear regression modelling for prediction of year score percentage^*^

Independent variable	β	SE	p-value^†^
ALSI Domain			
Surface Approach	- 0.16	0.16	0.005
Deep Approach	0.06	0.13	0.38
Effort management/Organised study	0.23	0.15	0.001
Monitoring studying	0.04	0.20	0.60
Time management^‡^	- 2.30	0.92	0.01
Social media use during study^§^	- 0.06	0.71	0.23

^*^Model fit: R² = 0.19, F = 8.71, p < 0.001; ^†^Adjusted for age, sex, year of programme, type of programme; ^‡^Categorical variable, two levels: “consistent”, “cramming” time management strategy; ^§^Categorical variable: never, sometimes, and often. β denotes the standardised variable estimate; SE denotes the standard error of the estimate.

### Study habits

A pertinent finding was the significant proportion (47.1%) of students relying on last minute “cramming” sessions to pass their examinations. Additionally, regression analysis revealed that poor time management was associated with decreased academic success in this sample. This suggests that almost half of the student body, regardless of mode of entry into medicine, fails to efficiently manage study time and spread the workload evenly throughout the academic year. In relation to undergraduate-entry students, it is possible that, as has been demonstrated in previous studies, ^24^^,^^33^ school-leavers are not equipped with the ability to manage their workload in a less structured university learning environment. If this is the case, remedial programmes to improve study skills and time management amongst incoming students should be considered an important element of all entry-level orientation programmes.^25^ Among GEM students, it may in part reflect over-generalising previously effective learning study habits while addressing new content in the new learning environment. Essentially, a “cramming” time management strategy may have proven useful during completion of their first degree, and the change in learning environment alone may not be a strong motivator for students to change formerly successful study habits.^34^

Our findings regarding OSN during study time are partially consistent with previous studies which have suggested a correlation between OSN use and poor academic performance.^29^^,^^31^ Although we failed to report any direct effect of using OSN sites during study time in our measure of academic success, we did identify that students who displayed poor, ineffective time management habits (“cramming” respondents) also tended to spend more time engaged in OSN during study time. As reported in a previous study,^35^ excessive use of OSN may contribute to a number of unhealthy behaviours/habits which are not conducive to stable academic performance; these factors include disruption of sleeping patterns and dietary problems,^35^ and the present study has demonstrated that poor time management is another such factor.

### Learning strategies

Our study further validates the use of the four-factor ALSI in medical students, as demonstrated by Mattick et al.^13^ With regard to the internal reliability of each factor, the Cronbach’s α values were acceptable for the deep approach (0.71), surface approach (0.64) and effort management/organised studying factors (0.74). However, the value for the monitoring study factor (0.58) was lower than values previously reported.^13^ The present sample scored relatively highly on the deep approach, monitoring studying and effort management/organised studying questions; mean numerical scores for each of these factors were all greater than 60% of their maximum possible score. Conversely, students reported lower scores in the surface approach category, with the mean numerical score being 46.05% of the maximum possible score.

The correlational analysis demonstrated a positive relationship between academic success and the effort management/organised studying factor. This factor corresponds with a more strategic learning approach described in educational literature, confirming that improving organisational study skills amongst medical students can lead to improved academic performance. The benefits of a strategic approach extend to increased flexibility, as students who adapt their learning approaches to the requirements and priorities of particular courses and testing modalities have a greater chance of academic success due to this flexible approach to learning.^37^ Use of the surface learning strategy was shown to be negatively correlated with year score percentage, confirming that this strategy is likely to contribute to a poor academic performance in medicine. It is widely assumed in medical education that the deep approach to learning is optimal, and that adopting a surface approach is associated with ineffective or temporary learning outcomes.^34^ However, some authors have noted that, traditionally, strategic or surface approaches to learning are common in undergraduate education, and that limiting opportunities for use of surface approaches is a recent feature of modern medical undergraduate curricula due to an increased emphasis on problem and case-based learning.^34^ In contrast to a previous study which shows that women are more likely to use the deep learning strategy,^16^ we found no statistical gender differences in any of the factors analysed in the ALSI.

The only statistically significant difference in study habits or learning strategies between UG and GEM students was found in scores for the surface learning strategy and monitoring studying factors. We found that UG students are more likely to adopt the surface learning strategy. This is unsurprising, as the UG students are predominantly school-leavers. In Ireland, the secondary-level exit examination (the ‘Leaving Certificate’) is highly structured, with predictable formats and recurrent patterns of questioning from year to year. This rewards pragmatic rehearsal of examination questions, as opposed to a deep vocational interest in the material. Students may become conditioned to engage in surface rote learning, and may continue to employ this undesirable strategy at university.

The principal limitation of the current study is that the main outcome measure, year score percentage, is a gross measure which does not take into account differences in assessment formats and content covered across the various year groups and programme types. Therefore, the possibility cannot be excluded that specific ALSI factors which were not found to be associated with year score percentage may however be more closely related to performance in specific areas in the undergraduate curriculum. 

## Conclusions

This study has yielded a number of significant results which are comparable with existing literature. It has highlighted to us that many medical students adopt unsuccessful study habits and learning strategies. This is likely to mirror the situation in other medical schools. Exploring students’ learning strategies can provide a substantiated framework on which to base any desired interventions or future research to rectify same, in medical education centres worldwide. With regard to learning strategies, the surface learning strategy should be avoided. The strategic approach should be fostered, in particular organisation of study and effort management. This work supports use of the ALSI as a useful screening tool to identify use of the pathological surface learning strategy. We suggest that it may be of heuristic value when assessing the efficacy of any initiative to improve knowledge and application of learning strategies, especially in the context of remediation of struggling students by academic mentors.

## Conflict of Interest

The authors declare that they have no conflict of interest.
